# Rich diversity and active spatial–temporal dynamics of *Thalassiosira* species revealed by time-series metabarcoding analysis

**DOI:** 10.1093/ismeco/ycad009

**Published:** 2024-01-10

**Authors:** Kuiyan Liu, Shuya Liu, Zongmei Cui, Yongfang Zhao, Nansheng Chen

**Affiliations:** CAS Key Laboratory of Marine Ecology and Environmental Sciences, Institute of Oceanology, Chinese Academy of Sciences, Qingdao 266071, China; Laboratory of Marine Ecology and Environmental Science, Qingdao National Laboratory for Marine Science and Technology, Qingdao 266200, China; College of Marine Science, University of Chinese Academy of Sciences, Beijing 100039, China; Center for Ocean Mega-Science, Chinese Academy of Sciences, Qingdao 266071, China; CAS Key Laboratory of Marine Ecology and Environmental Sciences, Institute of Oceanology, Chinese Academy of Sciences, Qingdao 266071, China; Laboratory of Marine Ecology and Environmental Science, Qingdao National Laboratory for Marine Science and Technology, Qingdao 266200, China; Center for Ocean Mega-Science, Chinese Academy of Sciences, Qingdao 266071, China; CAS Key Laboratory of Marine Ecology and Environmental Sciences, Institute of Oceanology, Chinese Academy of Sciences, Qingdao 266071, China; Laboratory of Marine Ecology and Environmental Science, Qingdao National Laboratory for Marine Science and Technology, Qingdao 266200, China; Center for Ocean Mega-Science, Chinese Academy of Sciences, Qingdao 266071, China; Laboratory of Marine Ecology and Environmental Science, Qingdao National Laboratory for Marine Science and Technology, Qingdao 266200, China; Center for Ocean Mega-Science, Chinese Academy of Sciences, Qingdao 266071, China; Jiaozhou Bay National Marine Ecosystem Research Station, Institute of Oceanology, Chinese Academy of Sciences, Qingdao 266071, China; CAS Key Laboratory of Marine Ecology and Environmental Sciences, Institute of Oceanology, Chinese Academy of Sciences, Qingdao 266071, China; Laboratory of Marine Ecology and Environmental Science, Qingdao National Laboratory for Marine Science and Technology, Qingdao 266200, China; Center for Ocean Mega-Science, Chinese Academy of Sciences, Qingdao 266071, China; Department of Molecular Biology and Biochemistry, Simon Fraser University, 8888 University Drive, Burnaby, British Columbia V5A 1S6, Canada

**Keywords:** *Thalassiosira*, metabarcoding analysis, Jiaozhou Bay, harmful algal bloom, spatial–temporal dynamics

## Abstract

*Thalassiosira* is a species-rich genus in *Bacillariophyta* that not only contributes positively as primary producer, but also poses negative impacts on ecosystems by causing harmful algal blooms. Although taxonomical studies have identified a large number of *Thalassiosira* species, however, the composition of *Thalassiosira* species and their geographical distribution in marine ecosystems were not well understood due primarily to the lack of resolution of morphology-based approaches used previously in ecological expeditions. In this study, we systematically analyzed the composition and spatial–temporal dynamic distributions of *Thalassiosira* in the model marine ecosystem Jiaozhou Bay by applying metabarcoding analysis. Through analyzing samples collected monthly from 12 sampling sites, 14 *Thalassiosira* species were identified, including five species that were not previously reported in Jiaozhou Bay, demonstrating the resolution and effectiveness of metabarcoding analysis in ecological research. Many *Thalassiosira* species showed prominent temporal preferences in Jiaozhou Bay, with some displaying spring–winter preference represented by *Thalassiosira tenera*, while others displaying summer–autumn preference represented by *Thalassiosira lundiana* and *Thalassiosira minuscula*, indicating that the temperature is an important driving factor in the temporal dynamics. The application of metabarcoding analysis, equipped with appropriate molecular markers with high resolution and high specificity and databases of reference molecular marker sequences for potential all *Thalassiosira* species, will revolutionize ecological research of *Thalassiosira* species in Jiaozhou Bay and other marine ecosystems.

## Introduction

The diatom genus *Thalassiosira* of the family *Thalassiosiraceae*, order *Thalassiosirales*, class *Mediophyceae*, and phylum *Bacillariophyta* [1, 2] represents an important component of the phytoplankton community that contributes significantly to marine primary productivity [3]. As such, some *Thalassiosira* species including *Thalassiosira weissflogii* have been employed as water quality indicators [4, 5]. Despite their many important positive contributions to the marine ecosystems [5], some *Thalassiosira* species can also pose significant negative impact on marine ecosystems by developing harmful algal blooms (HABs) [6, 7].

*Thalassiosira* is a species-rich genus with 179 officially accepted species, together with 19 accepted varieties and one accepted forma based on AlgaeBase [8]. In China, taxonomical studies have described 75 *Thalassiosira* species, together with eight *Thalassiosira* varieties and one *Thalassiosira* conformis, notably with many new records of *Thalassiosira* species identified in different ocean regions [9-13]. For example, four *Thalassiosira* species were identified based on morphological features in the Dapeng Bay, South China Sea [14]. Twenty-four *Thalassiosira* species, including three new *Thalassiosira* records, were identified in water samples collected from Zhejiang coast of the East China Sea [15]. New *Thalassiosira* species have also been frequently uncovered in China. For example, *Thalassiosira sinica* was first identified in the South China Sea and described based on morphological observations and phylogenetic analyses [16].

Among all *Thalassiosira* species, 11 are annotated as HAB species, including *Thalassiosira curviseriata*, *Thalassiosira decipiens*, *Thalassiosira diporocyclus*, *Thalassiosira excentrica*, *Thalassiosira hyalina*, *Thalassiosira mala*, *Thalassiosira minuscula*, *Thalassiosira nordenskioeldii*, *Thalassiosira pacifica*, *Thalassiosira rotula*, *Thalassiosira subtilis*, and *T. weissflogii*, all of which have also been identified in coastal regions of China [6, 8, 12, 17, 18], suggesting that *Thalassiosira* HAB species are likely cosmopolitan. Among these 11 *Thalassiosira* HAB species, eight have been reported to cause HABs with negative impacts on coastal waters of China, with *T. rotula* being the most frequent *Thalassiosira* HAB species in China [6].

In contrast to these large number of *Thalassiosira* species (75 species, 8 varieties, and 1 conformis) characterized in taxonomical studies, many fewer *Thalassiosira* species (43 species and 2 varieties) have been identified in ecological studies carried out in the coastal regions in China, despite hundreds of such ecological studies having been carried out in these regions over the last ~40 years [19-24]. In one such ecological study carried out in the northern South China Sea in the winter of 2004, only seven *Thalassiosira* species were identified among 198 phytoplankton described [25].

The discrepancy between numbers of *Thalassiosira* species identified in taxonomical studies and in ecological studies was largely attributed to the different methodologies applied in these two conditions. Although ecological studies often identify *Thalassiosira* species by almost exclusively examining morphological features using light microscopy (LM) [25, 26], taxonomists routinely apply a whole range of approaches including LM, scanning electron microscopy, and molecular analyses [16, 27]. Morphological features of *Thalassiosira* species, like many other diatom species, are not only diverse [28, 29], but also plastic [30-32], making it extremely challenging to annotate *Thalassiosira* species with adequate accuracy via LM observations alone.

Metabarcoding analysis approaches have been demonstrated to be effective to accurately map DNA sequences to taxonomic information [33, 34]. For example, one study identified 15 *Thalassiosira* species by applying metabarcoding analysis of 18S rDNA V4 regions of samples collected from Beaufort Sea sea-ice samples and water-column samples collected from the Canadian Arctic and the Scotia Shelf, North Atlantic, and identified two distinct *Thalassiosirales* communities [35]. Because of the high resolution, metabarcoding analysis can also be used to resolve spatial–temporal dynamics of *Thalassiosira* species. For example, through conducting metabarcoding analyses using 18S rDNA V4 regions in the Western English Channel (SOMLIT-Astan station), nano-species from the genus *Thalassiosira* were found to be the key components of the phytoplankton community in coastal waters. Different *Thalassiosira* species were found to show differential seasonal preferences, with *T. curviseriata* occurring mainly in spring, while *Thalassiosira* cf. *profunda* blooming during winter [18].

**Figure 1:**
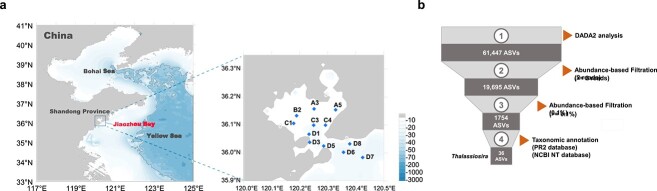
Illustration of sampling sites in Jiaozhou Bay and analysis procedure; (A) locations of 12 sampling sites (diamond) in Jiaozhou Bay, located in the Yellow Sea of Shandong Province, China; (B) four-step filtration and annotation procedure; numbers of ASVs were included for each step.

Many *Thalassiosira* species showed strong seasonal preferences, reflecting the influence of a combination of environmental factors, including temperature, large-scale climatic processes, grazing by zooplankton, and spore formation [36], with temperature being identified as the primary factor [37, 38]. *T. nordenskioeldii*, for example, is considered to be a cold-water species [39], has been observed in winter and spring at 0.5–8.5°C [36, 38], and has been found to cause spring blooms [40, 41]. *T. curviseriata* has also been identified mainly during spring (late March to early June) and found to cause spring blooms [12, 18]. In contrast, *T. minuscula* has been considered as a warm-water species [3, 40, 42-44]. Still some *Thalassiosira* species have been found to develop resting stages [45] that allow facilitate the survival of these algae under adverse conditions [46] including resting cells for *T. nordenskioeldii* and *T. rotula* [47, 48] or resting spores for *Thalassiosira antarctica*, *Thalassiosira australis*, *Thalassiosira constricta*, *Thalassiosira gravida*, and *Thalassiosira scotia* [49-53]. The development of resting cells and resting spores may be coincided with the decline of *Thalassiosira* cells in water. With the fluctuation of water temperature, resting stages may play an important role in seasonal distribution of *Thalassiosira* cells in water [36]. With the application of metabarcoding analysis and more extensive sampling, we expect to track known *Thalassiosira* species, identify new *Thalassiosira* species, and uncover the seasonal preference of these *Thalassiosira* species with much improved resolution.

Jiaozhou Bay is a semi-enclosed coastal bay in the Yellow Sea of China (Fig. 1A) [54]. As a model microcosm of coastal ecosystems, long-term and synchronous studies of phytoplankton dynamic distributions have been carried out, yielding valuable datasets for understanding the structure, function, and trends of the entire coastal ecosystem of China [23, 26, 55] (Supplementary Table 1). Twenty-seven *Thalassiosira* species (including one variety) in Jiaozhou Bay (Fig. 1C, blue) have been reported by different groups using different approaches. Qian and colleagues [56] identified five *Thalassiosira* species from net samples collected in Jiaozhou Bay. Li [10] identified 13 *Thalassiosira* species in monthly water samples collected from April 2005 to May 2006. Yang and colleagues identified five *Thalassiosira* species from water samples by means of microscopic examination [57]. Almost all the *Thalassiosira* HAB species except *T. minuscula* have been identified in Jiaozhou Bay, suggesting that Jiaozhou Bay is an excellent ocean region for *Thalassiosira* HAB research. Although *T. nordenskioeldii* and *T. rotula* were most frequently identified HAB species in Jiaozhou Bay, *T. nordenskioeldii* was the only *Thalassiosira* species that has been reported to cause HABs in Jiaozhou Bay [58, 59]. In a metabarcoding analysis of phytoplankton samples collected in a single expedition from Jiaozhou Bay, *Thalassiosira* species that were not reported in Jiaozhou Bay previously were identified, including *Thalassiosira concaviuscula*, *Thalassiosira minicosmica*, and *Thalassiosira punctigera*, demonstrating the strength of metabarcoding analysis of *Thalassiosira* species [60] and suggesting that more *Thalassiosira* species exist in Jiaozhou Bay. We hypothesize that the diversity and spatiotemporal of *Thalassiosira* species in Jiaozhou Bay can be better ascertained by the use of metabarcoding analysis.

In this study, we analyzed a set of time-series samples collected at 12 sampling sites monthly in a full year (from January to December, 2019) in Jiaozhou Bay by applying metabarcoding analysis using 18S rDNA V4 region as the barcode, with the aim to understand *Thalassiosira* species diversity and spatial–temporal dynamic distribution. We also explored interactions between species and the influence of environmental factors.

## Material and methods

### Study site and sample collection

Water samples were collected monthly at 12 sampling sites in Jiaozhou Bay, including four sampling sites inside the bay (A3, A5, B2, and C1), five sampling sites at the mouth of the bay (C3, C4, D1, D3, and D5), and three sampling sites outside the bay (D6, D7, and D8) from January 2019 to December 2019 (Fig.1A). Samples were collected onboard the research vessel “Chuangxin” operated by Jiaozhou Bay National Marine Ecosystem Research Station. Details of sample collection and treatment were described previously [23, 60]. Briefly, at each sampling site, 1 L seawater sample was collected, followed by removing large-sized plankton using 200 μm meshes (Hebei Anping Wire Mesh Co., Ltd., Hengshui, China). Because some *Thalassiosira* species are found alone as single cells, while others form chains of cells that have a wide size range, and cells of different size ranges may represent different *Thalassiosira* species. To explore this possibility, seawater samples were successively filtered through 10 and 0.2 μm filter polycarbonate membranes (Millipore, Burlington, MA) into two size fractions, 10–200 μm and 0.2–10 μm.

Environmental factor data were provided by Jiaozhou Bay National Marine Ecosystem Research Station, Institute of Oceanology, Chinese Academy of Sciences. Briefly, temperature and salinity were obtained using conductivity–temperature–depth sensor (ALEC ASTD-102) (JFE Advantech Co., Tokyo, Japan), concentrations of important nutrients including silicate ion (SiO_3_^2−^), phosphate ion (PO_4_^3−^), nitrite ion (NO_2_^−^), nitrate ion (NO_3_^−^), ammonium ion (NH_4_^+^), and dissolved inorganic nitrogen (DIN) (NO_3_^−^ + NO_2_^−^ + NH_4_^+^) were obtained using an autoanalyzer (Skalar San++, Breda, Netherlands), and chlorophyll concentrations were measured using the Turner-Designs Trilogy™ laboratory fluorometer (Turner, California) [61].

### Deoxyribonucleic Acid extraction, amplification, and sequencing

Details of DNA extraction and PCR amplification were described previously [62, 63]. Briefly, DNAs were extracted from filter membrane samples collected from water samples using the HP Plant DNA Kit (Omega, Norwalk, CT) according to the manufacturer’s protocols. The 18S rDNA V4 regions were amplified with TAReuk454FWD1 (5′-CCAGCA(G/C)C(C/T)GCGGTAATTCC-3′) and TAReukREV3 (5′-ACTTTCGTTCTTGAT(C/T)(A/G)A-3′) primers [62] with a unique barcode to distinguish samples, following PCR procedure described previously [63]: initial denaturation at 94°C for 4 min, followed by 32 amplification cycles of 1 min at 94°C, 1 min annealing at 57°C, and 2 min extension at 72°C with a terminal cycle of 10 min at 72°C. The PCR products were detected by electrophoresis with 1% agarose gels and sequenced on the Illumina NovaSeq platform (Illumina, San Diego, CA; Novogene, Beijing, China) with paired-end reads of 250 bp in length.

### Bioinformatics analysis

DADA2 in R was used to analyze the sequencing results [64, 65], with the following parameters from [60]: maxEE = c (2, 2), minLen = 200, truncLen = c (220, 220), minBoot = 80, and Min overlap = 12 bases. To retain the same sequencing depth, we performed rarefaction by subsampling each sample to the lowest number of reads per sample (48 274 reads), reserving a total of 61 447 amplicon sequence variants (ASVs).

ASVs were filtered and annotated in three steps (Fig. 1B): (i) ASVs supported by two or more reads in at least one sample were selected for further analysis to reduce the sequencing errors; (ii) only ASVs whose relative abundance was higher than 0.1% of the total abundance of at least one sample were used in subsequent analyses; (3) ASVs were preliminarily annotated to the genus level using the assignTaxonomy function with a threshold of 80% bootstrap confidence in the protist ribosomal reference database [66]. ASVs that were not annotated to any species in the previous step were further annotated using BLAST against the National Center for Biotechnology Information (NCBI) NT database, with a percentage identity threshold of 99% and coverage threshold of 95%. Based on the above two-step taxonomic annotation, all ASVs corresponding to the genus *Thalassiosira* were retained for subsequent spatial–temporal dynamics and diversity analysis.

### Statistical analysis

Spatial–temporal dynamics and diversity analysis of *Thalassiosira* ASVs in Jiaozhou Bay were carried out with reads number representing relative abundance. Haplotype networks were constructed using the TCS algorithm [67] in PopART v. 1.7 [68]. Upset plots were created based on surface seawater samples data using the R package UpSetR [69]. Community α diversities were analyzed by calculating richness, the Chao1 index, the Abundance-based Coverage Estimator (ACE) index, the Simpson diversity index, the Shannon–Wiener diversity index, and the Pielou evenness index based on surface seawater samples data using the picante package [70] in R and visualized with the interactive platform GraphPad Prism v9 (GraphPad Software, USA). The abundances of the ASVs count were subjected to centered log-ratio (CLR) transformation using the compositions package [71] in R to compensate for the constraints inherent to working with compositional data [72]. Community β diversities were analyzed through the compositional principal component analysis (PCA) based on a Euclidean distance matrix derived from CLR-transformed data using the coda.base package [73] in R. The differences of each sample between seasons and between sampling regions were statistically tested with permutational multivariate analysis of variance ) using the vegan package [74] in R. Phylogenetic tree on the left of heat map based on the 18S rDNA V4 region sequences was obtained using the maximum likelihood (ML) with 1000 bootstraps in MEGA X [75]. Heat maps were plotted based on surface seawater samples data using the pheatmap package [76] in R. Sampling sites and spatial distributions of ASV_270 and ASV_29 were drawn based on surface seawater samples data using Surfer 16 program (Golden Software LLC, USA). Correlation analyses between environmental factors and *Thalassiosira* ASVs abundance were carried out using the corrplot package [77] in R, and between other ASVs and *Thalassiosira* ASVs abundance was carried out using the psych package [78] in R. Correlation networks were plotted using Cytoscape [79].

**Figure 2:**
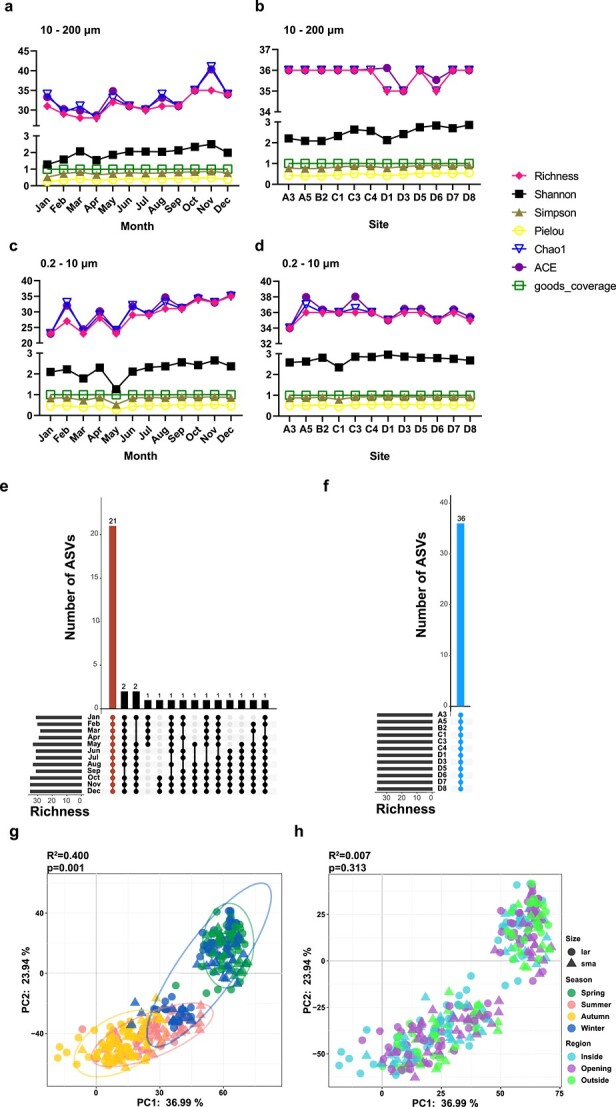
Community diversity analysis of *Thalassiosira* in surface seawater samples; alpha diversity analysis of 36 *Thalassiosira* ASVs based on multiple indices (A) for monthly variation at 10–200 μm size, (B) for variation among 12 sites at 10–200 μm size, (C) for monthly variation at 0.2–10 μm size, (D) for variation among 12 sites at 0.2–10 μm size; upset maps show the distribution and richness of the 36 *Thalassiosira* ASVs in different samples, including (E) at 12 months and (F) at 12 sites; three parts of the upset maps: the upper part shows the intersection size, which means the number of ASVs shared by each month or station; the lower left part shows the number of elements in each set, which means the ASV richness in each month or station, and the lower right part shows the intersection relation of sets, that is, the connection of points represents the ASVs sharing status of each month or station; beta diversity analysis of 36 *Thalassiosira* ASVs using PCA method (G) at season (95% confidence interval), and (H) region.

## Results

### *Thalassiosira* diversity detected in Jiaozhou Bay through metabarcoding analysis

Among 1754 ASVs obtained in the DADA2 analysis of the samples collected in Jiaozhou Bay (Fig. 1A), 36 ASVs were annotated to represent *Thalassiosira* species (Fig. 1B). Thirty-six *Thalassiosira* ASVs richness and diversity were slightly higher from June to November than those from January to May (Fig. 2A, C, and E). During the time course of this project (January 2019–December 2019), many *Thalassiosira* ASVs showed strong temporal preferences, with only about half of ASVs were shared for 12 months throughout the year (Fig. 2E, red). In contrast, all 36 *Thalassiosira* ASVs were found at each of the 12 sites (Fig. 2B, D, and F, blue), suggesting a lack of spatial preference among different sampling sites.

PCA plot of *Thalassiosira* ASVs in surface seawaters according to sampling seasons and sampling regions (inside the bay, bay mouth, and outside the bay) showed that *Thalassiosira* ASVs had similar community structures in winter and spring, and similar community structures in summer and autumn (Fig. 2G). In contrast, ASVs of different cell sizes did not show much difference in community structures. Also, the community structures were similar among different regions of Jiaozhou Bay (Fig. 2H).

**Figure 3:**
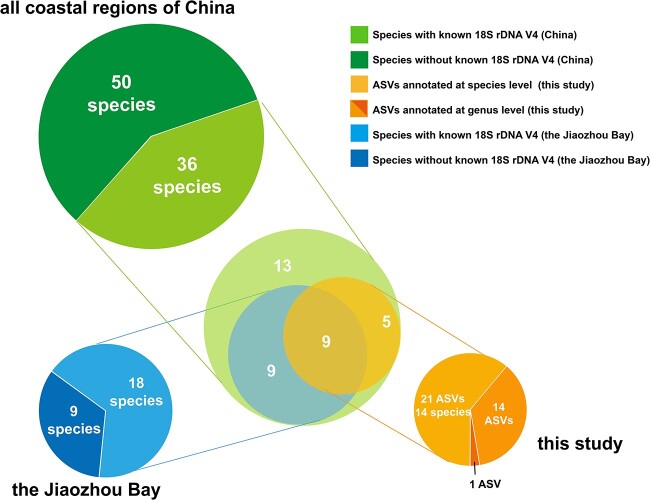
Comparison of the numbers of *Thalassiosira* species identified in this study with the number of *Thalassiosira* species reported previously in Jiaozhou Bay and in the coastal regions of China.

Among these 36 *Thalassiosira* ASVs (Table 1), 21 ASVs were annotated as 14 known *Thalassiosira* species (Fig. 3, light orange). Among these 14 *Thalassiosira* species, three were annotated as HAB species, including *T. mala*, *T. minuscula*, and *T. rotula* [6, 8, 12, 17, 18]. In addition, 15 *Thalassiosira* ASVs (Fig. 3, orange and deep orange) represented previously unannotated *Thalassiosira* species. Some of these ASVs each corresponded to multiple species (Fig. 3, orange), indicating that multiple *Thalassiosira* species share an identical 18S rDNA V4 sequence, suggesting that the molecular marker 18S rDNA V4 did not have adequate resolution to resolve these *Thalassiosira* species. Among the 86 *Thalassiosira* species previously identified in coastal waters of China (Fig. 3, light green and green), sequences of the molecular marker 18S rDNA V4 were available for only 36 species (Fig. 3, light green). Of these 14 *Thalassiosira* species (Fig. 3, light orange), nine have been previously identified in Jiaozhou Bay. Among the 27 *Thalassiosira* species identified previously in Jiaozhou Bay (Fig. 3, light blue and blue; Supplementary Table 1), 18 did not appear in the annotation of the dataset in this study, including nine that had low abundance, and nine whose 18S rDNA V4 information was not available (Fig. 3, blue).

**Table 1:** Annotated list of *Thalassiosira* ASVs in Jiaozhou Bay in this study.

**ASV_name**	**ASV_length/bp**	**Group** ^**a**^	**Species**	**Accession_number**	**Identity%**	**HAB**
ASV_11	384	1vm	*T. aestivalis/T. pacifica*	DQ514873/DQ514888	100	
ASV_29	384	mv1	*T. concaviuscula*	AJ810857	99.74	Non-HAB
ASV_30	384	1vm	*T. curviseriata/T. profunda*	KJ671713/AM235383	100	
ASV_39	384	1vm	*T. proschkinae/Minidiscus spinulatus*	KY912618/MN179316	100	
ASV_55	383	1v1	*T. minima*	DQ514876	100	Non-HAB
ASV_62	384	1v1	*T. rotula*	AF374480	100	HAB
ASV_64	384	mv1	*T. concaviuscula*	AJ810857	100	Non-HAB
ASV_76	384	1v1	*T. oceanica*	KY980420	100	Non-HAB
ASV_83	383	1vm	*T. minima/T. nordenskioeldii*	JN934676/DQ514886	100	
ASV_99	383	1v1	*T. mala*	HM991693	100	HAB
ASV_108	384	1vm	*T. allenii/T. angulata*	GU823604/DQ514867	100	
ASV_119	384	1v1	*T. tenera*	HM991701	100	Non-HAB
ASV_131	384	1vm	*T. angulata/T. anguste-lineata*	KF454006/AJ810854	99.479	
ASV_132	384	1vm	*T. bioculata/Minidiscus trioculatus/T. oestrupii var. venrickae*	OK147671/HQ912563/DQ514870	99.74	
ASV_139	384	1v1	*T. lundiana*	MG905326	100	Non-HAB
ASV_143	384	mv1	*T. profunda*	MW205689	100	Non-HAB
ASV_172	384	1vm	*T. eccentrica/T. minima*	MG905325/FR865522	99.219	
ASV_208	384	1vm	*T. rotula/T. tenera*	KJ671715/MW722948/OK147676	99.219	
ASV_270	384	mv1	*T. minuscula*	DQ514887	99.479	HAB
ASV_280	384	1v1	*T. oestrupii var. venrickae*	DQ514870	100	Non-HAB
ASV_292	384	mv1	*T. profunda*	KC284713	99.74	Non-HAB
ASV_347	384	1vm	*T. eccentrica/T. minima*	JQ781887/DQ514868/FR865522	100	
ASV_353	384	1v1	*T. minicosmica*	MF405352	100	Non-HAB
ASV_406	384	mv1	*T. minuscula*	DQ514882	100	HAB
ASV_410	384	mv1	*T. concaviuscula*	HM991689	100	Non-HAB
ASV_606	384	genus level	*Thalassiosira* sp.			
ASV_631	384	mv1	*T. concaviuscula*	AJ810857	99.219	Non-HAB
ASV_643	384	mv1	*T. profunda*	KC284713	100	Non-HAB
ASV_731	384	1v1	*T. punctigera*	AJ810856	100	Non-HAB
ASV_860	384	mv1	*T. concaviuscula*	AJ810857	99.479	Non-HAB
ASV_1262	384	1vm	*T. angulata/T. anguste-lineata*	KF454006/AJ810854	99.74	
ASV_1305	384	1v1	*T. andamanica*	MF405350	99.219	Non-HAB
ASV_1868	384	1v1	*T. secreta*	MK376464	99.221	Non-HAB
ASV_2261	384	1vm	*T. pacifica/T. aestivalis*	HM991697/DQ514873	100	
ASV_3005	384	1vm	*T. tenera/Bacterosira constricta/Bacterosira bathyomphala*	KY980238/KT692951/DQ514894	99.479	
ASV_3070	384	1vm	*T. allenii/T. angulata*	HM991688/DQ514867	99.74	

^a^1v1: one ASV corresponded to one definite *Thalassiosira* species mv1: multiple ASVs corresponded to one definite *Thalassiosira* species 1vm: one ASV corresponding to multiple species genus level: one ASV did not annotated as any definite *Thalassiosira* species

**Figure 4:**
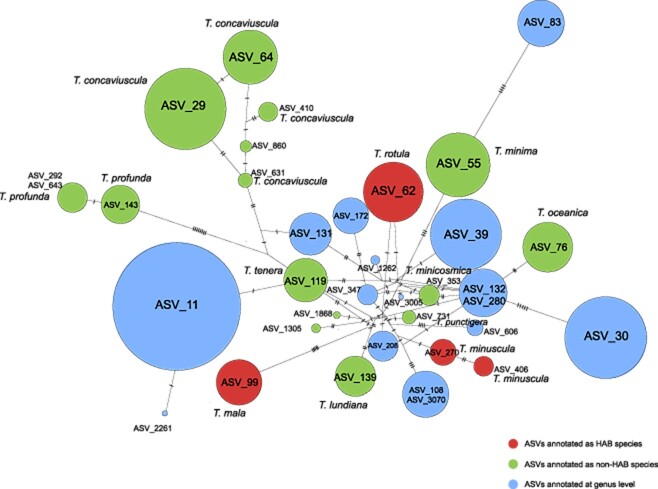
TCS network of *Thalassiosira* ASVs in Jiaozhou Bay; three categories of *Thalassiosira* ASVs were displayed, representing HAB species, non-HAB species, and multiple species; the lines represented the evolutionary relationships between ASVs, and the number of short vertical lines represented the numbers of difference sites between the pairs of ASVs; circle sizes represented the relative abundance of ASVs. The species written in red were HAB species.

Some *Thalassiosira* species in Jiaozhou Bay were represented by multiple ASVs, suggesting that these *Thalassiosira* species may have rich intraspecific diversity. For example, five ASVs (ASV_29, ASV_64, ASV_410, ASV_631, and ASV_860) corresponded to *T. concaviuscula*, while three ASVs (ASV_143, ASV_292, and ASV_643) corresponded to *T. profunda* (Fig. 4). Six previously known HAB species were identified in Jiaozhou Bay, including *T. rotula* (ASV_62, ASV_208), *T. mala* (ASV_99), *T. minuscula* (ASV_270 and ASV_406), and possibly *T. pacifica* (ASV_11 and ASV_2261), *T. curviseriata* (ASV_30), and *T. nordenskioeldii* (ASV_83). Among all 11 known *Thalassiosira* HAB species, only *T. minuscula*, which is frequently reported from warm-water regions [3, 40, 42, 43], was not identified previously in Jiaozhou Bay. It has been suggested that the rare occurrence of *T. minuscula* may be related to the fragility of the valve wall and/or its dissolution in stationary liquid [80]. The successful identification of *T. minuscula* (ASV_270 and ASV_406) in this study further demonstrated the strength of DNA metabarcoding analysis.

### Spatial–temporal dynamic distribution of *Thalassiosira* in Jiaozhou Bay

**Figure 5:**
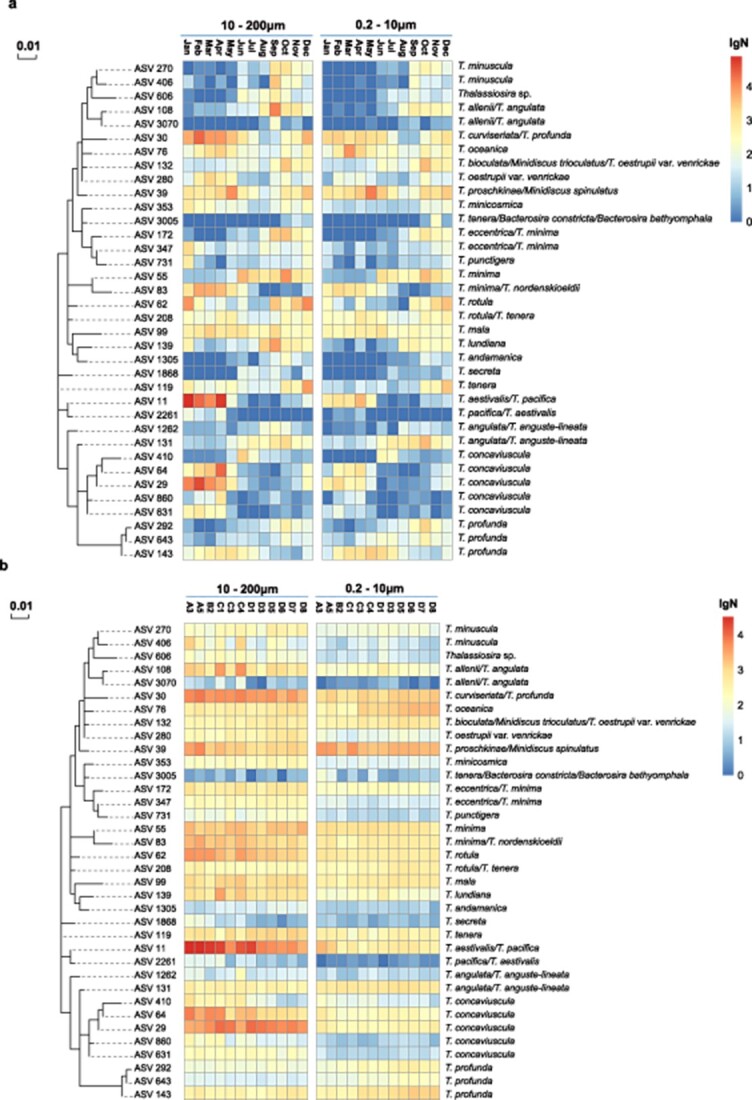
ML tree based on the sequences of 18S rDNA V4 region and (A) temporal distribution heat map and (B) spatial distribution heat map based on the relative abundance of *Thalassiosira* ASVs in surface seawater, including two cell size types, 10–200 μm and 0.2–10 μm; the species names on the right were annotation results for each *Thalassiosira* ASVs.

**Figure 6:**
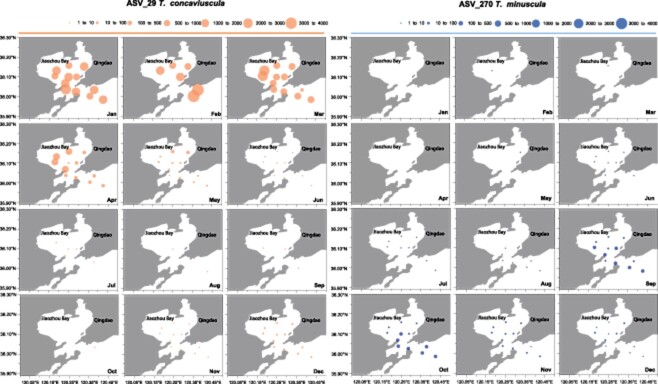
Spatial–temporal distribution of ASV_29 (*T. concaviuscula*) and ASV_270 (*T. minuscula*) in 12 sites and 12 months (January 2019–December 2019); the circle area showed the relative abundance of ASVs.

Relative abundance analysis revealed that many *Thalassiosira* ASVs showed strong temporal preferences during January 2019 to December 2019 (Fig. 5A). Although some *Thalassiosira* ASVs showed strong preference for warm temperatures (June to November), others showed strong preference for cold temperatures (January to May and December). For example, *T. minuscula*, that was represented by ASV_270 (Figs 5A and 6 ) and ASV_406 (Fig. 5A), was found primarily from September to December, which is consistent with previous reports that this species was recorded in autumn from Daya Bay and Hong Kong off the Guangdong coast and Helgoland in the German North Sea [40, 42]. Similarly, *Thalassiosira lundiana* (ASV_139) was found to be present from August to December in Jiaozhou Bay, which was consistent to previous report that it was a warm-water species [9]. Our analyses revealed that *T. concaviuscula* (ASV_29, ASV_64, ASV_607, and ASV_860) showed strong preference for cold temperatures (Figs 5A and 6), which was consistent to a previous report that *T. concaviuscula* were recorded mainly from October to April in the North Sea at Helgoland (German Bight) [40]. In addition to the above *Thalassiosira* species that show strong preference for warm or cold temperatures, some *Thalassiosira* species were cosmopolitan and showed eurythermic distribution, including *Thalassiosira oceanica* (ASV_76) and *T. mala* (ASV_99) [3, 40, 42, 81], both of which showed wide temporal distribution without strong temporal preference.

In contrast, none of these *Thalassiosira* ASVs showed strong spatial preference in Jiaozhou Bay (Fig. 5B).

### Correlation of *Thalassiosira* amplicon sequence variant abundance with environmental factors

**Figure 7:**
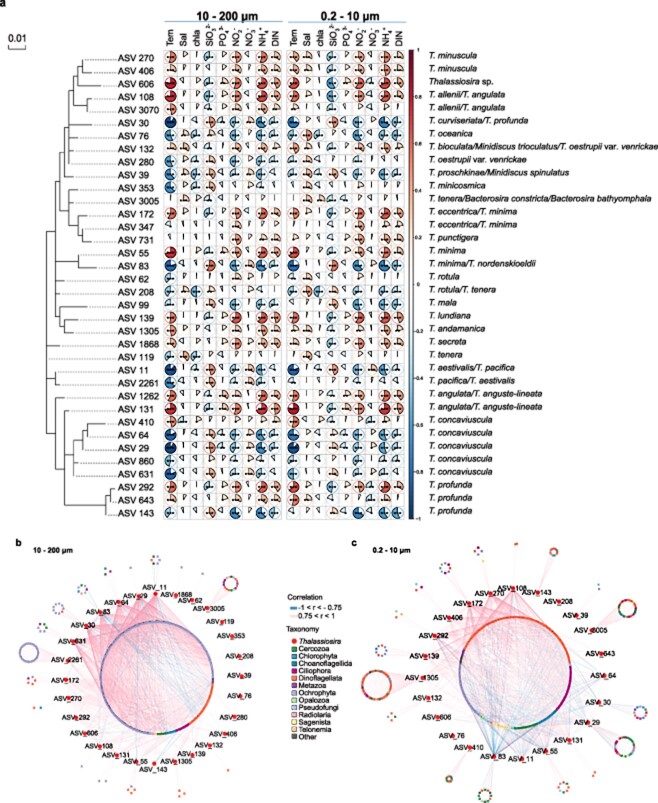
Correlation analysis of *Thalassiosira* ASVs abundance; (A) correlation with nine environmental factors, including temperature, salinity, concentration of chlorophyll a, SiO_3_^2−^, PO_4_^3−^, NO_2_^−^, NO_3_^−^, NH_4_^+^, and dissolved inorganic nitrogen (DIN); the solid area and color of the pie indicated the magnitude of correlation coefficient with * indicating that *P* < .05, with ** indicating that *P* < .01, with *** indicating that *P* < .001; (B) correlation with all 19 695 plankton ASVs, |r| > 0.75, *P* < .001, in the large size (10–200 μm); (C) correlation with all 19 695 plankton ASVs, |r| > 0.75, *P* < .001, in the small sizes (0.2–10 μm); *Thalassiosira* ASVs in this study were marked as dots in the middle layer, and the plankton ASVs were marked as squares in the outer circle and the inner circle.

To explore the impact of environmental factors, including temperature, salinity, concentration of chlorophyll a, SiO_3_^2−^, PO_4_^3−^, NO_2_^−^, NO_3_^−^, NH_4_^+^, and DIN on the *Thalassiosira* ASVs, correlation between the abundance of *Thalassiosira* ASVs and various environmental factors was analyzed (Fig. 7A). As expected, some ASV abundances were strongly positively correlated with temperature, including *Thalassiosira anguse-lineata* (ASV_131, ASV_1262), *Thalassiosira angulata* (ASV_131, ASV_1262, ASV_108, ASV_3070), and *T. minuscula* (ASV_270, ASV_406). On the other hand, other ASV abundances were strongly negatively correlated with temperature, including *T. oceanica* (ASV_76), *Thalassiosira aestivalis*/*T. pacifica* (ASV_11, ASV_2261) (Fig. 7A). Furthermore, we found that temperature could have differential impact on ASVs annotated as the same species. For example, the abundance of ASV_410, which was annotated as *T. concaviuscula* was strongly positively correlated with temperature, while ASV_29, ASV_64, ASV 631, and ASV_860, which were both annotated as *T. concaviuscula* were negatively correlated with temperature (Fig. 7A).

Correlation analysis of other environmental factors including concentration of NO_2_^−^, NH_4_^+^, and DIN revealed that these factors had similar impact on *Thalassiosira* ASVs abundance to that of temperature. While SiO_3_^2−^ and NO_3_^−^ had opposite impact on *Thalassiosira* ASVs abundance. Salinity, concentration of chlorophyll a, and PO_4_^3−^ were found to show weak correlation with the abundance of *Thalassiosira* ASVs. We also ascertained potential interactions between *Thalassiosira* species and other plankton species by conducting correlation analyses between the relative abundance of 36 *Thalassiosira* ASVs and that of all 19 695 plankton ASVs. For the large-sized cells (10–200 μm) (Fig. 7B), the abundance of 28 *Thalassiosira* ASVs showed overwhelmingly strong correlations with 420 plankton ASVs, dominated by *Ochrophyta* species (|r| > = 0.75, *P* < .01). Most correlations were positive (pink lines), which may reflect that during nonblooming, most species in *Ochrophyta* including diatom tend to have similar ecological preferences and thus exhibit similar distribution characteristics. Only a tiny fraction of the correlations between the abundance of the *Dinoflagellata* ASVs and *Thalassiosira* ASVs (blue lines) were negative. For the small sizes (0.2–10 μm) (Fig. 7C), a smaller number of strong correlations emerged: 23 *Thalassiosira* ASVs and 511 plankton ASVs that were dominated by *Dinoflagellata* species. Most of the correlations were also positive, with some *Thalassiosira* ASVs negatively correlated with the abundance of *Dinoflagellata* and *Ciliophora* ASVs, as could be explained by predator–prey relationship between some of *Dinoflagellata* or *Ciliophora* and smaller sized *Thalassiosira* species.

## Discussion

### Rich diversity of *Thalassiosira* species in Jiaozhou Bay

Metabarcoding analysis of monthly time-series samples collected in Jiaozhou Bay led to the identification of 14 known *Thalassiosira* species in Jiaozhou Bay. Among them, five *Thalassiosira* species were previously unannotated in Jiaozhou Bay, including *T. lundiana*, *Thalassiosira secreta*, *T. minuscula*, *Thalassiosira oestrupii var. venrickae*, and *Thalassiosira andamanica*. Among them, *T. lundiana* is morphologically similar to *T. punctigera* [9], making it challenging to distinguish these two species in previous investigations and the lack of report on *T. lundiana*. In addition, the variable external tube of labiate processes of *T. minuscula* [82] also add challenges to morphological identification of *Thalassiosira* species. Of the nine *Thalassiosira* species previously recorded in Jiaozhou Bay that were not identified in this study, five of them were missed in this study because their 18S rDNA V4 reference sequences were identical to that of other species and could not be distinguished. Of the remaining four species, *Thalassiosira pseudonana* and *T. weissflogii* were not identified because they have been transferred to other genera *Cyclotella* and *Conticribra* [83, 84], corresponding to *Cyclotella nana* (ASV_4297) and *Conticribra weissflogii* (ASV_14058 and ASV_44057), respectively. Therefore, they no longer belong to genus *Thalassiosira* and were thus excluded from the identification list in this study during the ASV filtrations. Both of them were also detected but with lower relative abundance in this study. The above analysis revealed high *Thalassiosira* diversity with many previously unannotated *Thalassiosira* species in Jiaozhou Bay (Fig. 4**;**  Table 1). Although many ASVs corresponded to known *Thalassiosira* species (Fig. 4, green and red circles), many ASVs each corresponded to multiple *Thalassiosira* species, again suggesting that 18S rDNA V4 was inadequate for resolving these species (Fig. 4, blue circles).

Two ASVs (ASV_11 and ASV_2261), which were annotated as *T. pacifica*/*T. aestivalis* because these two species shared identical 18S rDNA V4 sequences, both ASVs showed much higher relative abundances in spring and winter than that in summer and autumn. As previous studies show that *T. aestivalis*, but not *T. pacifica* showed preference for spring and winter [36], these two ASVs most likely represent *T. aestivalis*, which often co-exists with *T. concaviuscula* [40]. *Thalassiosira* species can exist as single cells and can form chains with many cells, analysis showed that *Thalassiosira* ASVs derived from samples obtained using different filtrations (0.2–10 μm and 10–200 μm) had similar spatial–temporal distribution characteristics (Fig. 5).

### Strong temporal preferences of *Thalassiosira*

This project revealed that many *Thalassiosira* species identified in Jiaozhou Bay showed high differential temporal preferences, which can be generally divided into two groups: a spring–winter group represented by *T. tenera* and a summer–autumn group represented by *T. lundiana* and *T. minuscula*. Temporal preferences by different *Thalassiosira* species have previously been reported, such as the spring preference of *T. profunda* and the summer preference of *T. pseudonana* in Jiaozhou Bay in China [10], and the spring preference of *T. nordenskioeldii* and the winter preference of *T. tenera* in the North Sea in Germany [40]. The co-existence of two groups of *Thalassiosira* species with complementary temporal preferences in an ecosystem may be the result of the complementary evolution of *Thalassiosira* species to adapt to different ecosystems [85, 86]. More strikingly, we found that some *Thalassiosira* species may develop intra-species differences in temporal preferences. While *T. profunda* (represented by ASV_292 and ASV_643) showed a winter preference, another variant of *T. profunda* (represented by ASV_143) showed summer preference. ASV_143 and ASV_292 differ from ASV_643 by one base at nucleotide position 97 and 93, respectively. Further investigations are needed to uncover molecular mechanisms underlying such temporal differences.

### Temperature as an important driving factor in the temporal dynamics

Our analysis demonstrated that, among many environmental factors, temperature appeared to be a decisive factor that regulates the abundance of *Thalassiosira* species. Metabarcoding analysis revealed that many species including *T. anguse-lineata* (ASV_131 and ASV_1262), *T. angulata* (ASV_131, ASV_1262, ASV_108, and ASV_3070), and *T. minuscula* (ASV_270 and ASV_406) were strongly positively correlated with temperature (Fig. 7A). These results were generally consistent with previous reports. For example, *T. minuscula* has been found to be more abundant in a warm-water whose relative abundance is higher with higher temperature [3, 40, 42-44].

In contrast, *Thalassiosira* species including *T. oceanica* (ASV_76), *T. aestivalis*/*T. pacifica* (ASV_11 andASV_2261) were strongly negatively correlated with temperature (Fig. 7A). Notably, metabarcoding analysis could distinguish temperature preferences between different strains of a same species, which could be differentially regulated by temperature. For example, the abundance of ASV_410 of *T. concaviuscula* was positively regulated by temperature, while ASV_29, ASV_64, ASV_631, and ASV_860 strains of *T. concaviuscula* were negatively regulated by temperature (Fig. 7A). These results were generally consistent with previous studies.

### Metabarcoding analysis serves as the next-generation methodology for ecological research on *Thalassiosira* species

Although much progress has been made in taxonomical research on the species-rich genus *Thalassiosira* with 199 *Thalassiosira* species annotated and confirmed internationally [8], and 75 *Thalassiosira* species, together with eight varieties and one conformis in China, only 43 *Thalassiosira* species and two varieties have been identified in ecological studies using primarily morphology-based studies [23, 25, 26], suggesting that the presence and relative abundance of a large number of *Thalassiosira* species remain elusive. Results from this study demonstrated the strength of metabarcoding analysis in not only identifying the presence of *Thalassiosira* species, but also their relative abundances in time-series samples, suggesting that metabarcoding analysis can be used as the next-generation methodology to accurately map the taxonomic information to environmental DNA sequences, so as to obtain ecological information [33].

However, to unleash the power of metabarcoding analysis for ecological research on *Thalassiosira* species, much effort needs to be made to achieve the accurate tracking of all *Thalassiosira* species. First, effort is needed to ensure accurate taxonomic definition of all *Thalassiosira* species. The morphological features among *Thalassiosira* species are diverse [28, 29] and plastic [30-32]. Subtle morphological differences are easily neglected, resulting in multiple species being identified as the same species [87], and one species can be identified as multiple species due to a variable and unstable morphological features [29]. As a result, taxonomic studies based on morphological and molecular characteristics of the *Thalassiosira sensu lato* have undergone constant revision and refinement. For example, taxonomists have proposed that the new genera *Roundia* [88], *Takanoa* [89], *Shionodiscus* [90], and *Conticribra* [83] should be separated from genus *Thalassiosira sensu lato*. And some *Thalassiosira* species may have been transplanted to other existing genera *Minidiscus* [27], *Cyclotella* [84], and *Planktoniella* [91, 92]. Similar revisions such as de-duplication of synonyms are still in progress, and taxonomic identifications of a large number of *Thalassiosira* species are still ambiguous [91]. Thus, accurate identification of *Thalassiosira* species using combined morphological and molecular features is urgently needed.

Second, effort is needed to select appropriate molecular markers for metabarcoding analysis. In this study, the sequences of many ASVs were found to be highly similar to multiple species (Table 1), making it impossible to annotate them to certain *Thalassiosira* species, suggesting that the resolution of 18S rDNA V4 region may not be sufficient for distinguishing all the *Thalassiosira* species. Therefore, efforts need to be made to evaluate the specificity of molecular markers of *Thalassiosira* species for metabarcoding analysis so that *Thalassiosira* species can be unambiguously identified. Preliminary comparison of the resolution of multiple molecular markers of 12 *Thalassiosira* species indicated that Internal Transcribed Space (ITS) had a higher resolution in the study of *Thalassiosira* species (Supplementary Tables 2 and 3). When more *Thalassiosira* species have been identified, further effort should be taken to evaluate both common molecular markers and new molecular markers for their utilization for metabarcoding analysis.

Third, effort is needed to ensure databases exist with complete molecular information for all *Thalassiosira* species. For example, of the 199 *Thalassiosira* species reported worldwide, 18S rDNA V4 sequences of only 42 *Thalassiosira* species have been reported (Supplementary Table 4). Among the 27 *Thalassiosira* species recorded in Jiaozhou Bay (Supplementary Table 1), nine of them have not had 18S rDNA V4 region sequence reported (Fig. 3, dark blue in the pie), though we note that the one genus-level ASV may correspond to some of these species. The lack of reference sequences was partly responsible for the inability to annotate many *Thalassiosira* ASVs in this study (Table 1). Therefore, completion of the databases is essential for the success of metabarcoding analysis. Once a more appropriate molecular marker is identified for metabarcoding analysis for *Thalassiosira*, reference sequences of this molecular marker for all *Thalassiosira* species need to be made available for metabarcoding analysis.

As for the 18S rDNA copy number, each species or even each cell varies in copy number and copy diversity. Currently, although the 18S rDNA copy number of some species can be estimated by real-time PCR and other methods [93], it is impossible to fully account for every species in the area. For more accurate instructions, our analysis was more likely to address relative abundance changes over different seasons or locations in specific *Thalassiosira* species, as opposed to fully quantifying abundance differences between different *Thalassiosira* species.

## Conclusion

Metabarcoding analysis of time-series samples collected in Jiaozhou Bay, China revealed high biodiversity of *Thalassiosira* species and striking temporal dynamic distribution. The competitive strength in identifying known *Thalassiosira* species and uncovering previously unannotated species illustrated that metabarcoding analysis combined with morphology-based methods can become a powerful tool in ecological studies of marine ecosystems. Further efforts need to be made to fully unleash the potential for metabarcoding analysis, including enhanced taxonomic definition of *Thalassiosira* species, selection of appropriate molecular markers for metabarcoding analysis, and the construction of a database with all reference sequences.

## Supplementary Material

Supplementary_Tables_ycad009

## Data Availability

The sequencing results (raw data) have been submitted to NCBI, and the BioProject numbers are PRJNA577777 and PRJNA733859.
